# Natural Products as Modulators of Aryl Hydrocarbon Receptor Signaling in Atopic Dermatitis Management

**DOI:** 10.3390/molecules29245951

**Published:** 2024-12-17

**Authors:** Jangho Lee, Hyo-Kyoung Choi, Hee Soon Shin, Gun-Dong Kim

**Affiliations:** 1 Division of Food Functionality Research, Korea Food Research Institute (KFRI), Wanju 55365, Republic of Korea; jhlee@kfri.re.kr (J.L.); chkyoung@kfri.re.kr (H.-K.C.); hsshin@kfri.re.kr (H.S.S.); 2 Department of Food Biotechnology, Korea University of Science and Technology (UST), Daejeon 34113, Republic of Korea

**Keywords:** atopic dermatitis, aryl hydrocarbon receptor, natural product, immune modulation, skin barrier function

## Abstract

Atopic dermatitis (AD) is a chronic inflammatory skin condition characterized by immune dysregulation, skin barrier dysfunction, and a significant patient burden. Recent studies have highlighted the aryl hydrocarbon receptor (AhR) as a promising therapeutic target for AD management because of its pivotal role in modulating immune responses and maintaining skin barrier integrity. The dysfunction of the AhR pathway has been linked to AD pathogenesis, emphasizing the need for therapies that can restore its regulatory functions. Natural products have emerged as potential modulators of the AhR and are effective and safe alternatives to conventional treatments. Compounds such as curcumin, resveratrol, quercetin, and microbial metabolites have demonstrated the ability to activate AhR, reduce inflammation, and promote skin barrier function. These natural agents have fewer side effects and enhance patient compliance compared with conventional therapies, making them attractive candidates for long-term AD management. The integration of natural products targeting the AhR pathway provides a multifaceted approach that alleviates symptoms, addresses underlying disease mechanisms, and promotes sustainable improvements in skin health. This review highlights the therapeutic potential of natural AhR modulators and their potential roles in enhancing patient outcomes through novel integrative treatment strategies.

## 1. Introduction

The aryl hydrocarbon receptor (AhR)-related signaling pathway has emerged as a promising target for alleviating the symptoms of atopic dermatitis (AD), a chronic inflammatory skin disease characterized by epidermal barrier dysfunction and immune dysregulation [1]. AhR is a ligand-activated transcription factor that plays a crucial role in maintaining skin barrier integrity and modulating immune responses. Upon ligand activation, AhR dissociated from the cytoplasmic complex formed with heat shock protein 90, hepatitis B virus X-associated protein 2, and p23, and translocates to the nucleus, then dimerizes with the AhR nuclear translocator and interacts with various transcriptional factors [2,3]. These processes are intricately and finely regulated for maintaining homeostasis. For instance, dioxins and polyaromatic hydrocarbons activate AhR via environmental ligands, leading to the production of reactive oxygen species and oxidative stress as well as promoting the generation of proinflammatory cytokines, including CYP1A1-mediated interleukin (IL)1, IL6, and IL8, thereby contributing to the pathogenesis of AD, psoriasis, skin cancer, and aging [4,5,6]. Dysregulation of the AhR-induced signaling pathways has been observed in AD, with increased expression of AhR and reduced activation of its downstream targets, such as CYP1A1, in lesional skin compared to healthy skin [7]. In contrast, tryptophan metabolites such as kynurenine present in the skin activate AhR through the endogenous ligands, upregulating the production of proteins associated with keratinocyte differentiation and skin barrier maintenance [8,9]. Additionally, AhR activation induces the nuclear factor-erythroid 2-related factor 2 (Nrf2) pathway, thereby enhancing the expression of antioxidant enzymes, including NAD(P)H dehydrogenase quinone 1, heme oxygenase-1, and glutathione S-transferase [10,11]. These contraverse regulatory effects are also observed in immune responses. AhR is highly expressed in innate lymphoid cells type 3 and Th17 cells, contributing to the skin inflammation mediated by the production of IL17 and IL22, whereas promotes the differentiation and function of regulatory T cells [12,13]. Natural products, including bioactive compounds such as quercetin, gallic acid, and ginsenosides, have shown the potential to modulate AhR-related signaling and exert anti-inflammatory effects, thereby offering a therapeutic avenue for AD management [14]. These compounds can reduce the activity of inflammatory cells and cytokines, which are pivotal in AD pathogenesis [14]. Furthermore, the interplay between AhR and other signaling pathways, such as IL13, has been highlighted, suggesting that AhR activation can attenuate IL13-induced responses, which are central to immune dysregulation in AD [1,15]. Tapinarof, a topical AhR agonist, has demonstrated efficacy in clinical trials for AD, underscoring the therapeutic potential of targeting this pathway [16]. Additionally, natural products have been explored for their ability to modulate skin and gut microbiota, which are implicated in AD pathogenesis, further supporting their role in comprehensive AD treatment strategies [17]. Overall, the integration of natural products with AhR signaling modulation presents a promising pathway for developing novel, safe, and effective treatments for AD [18].

## 2. Pathophysiology and Current Treatments of AD

**Figure 1:**
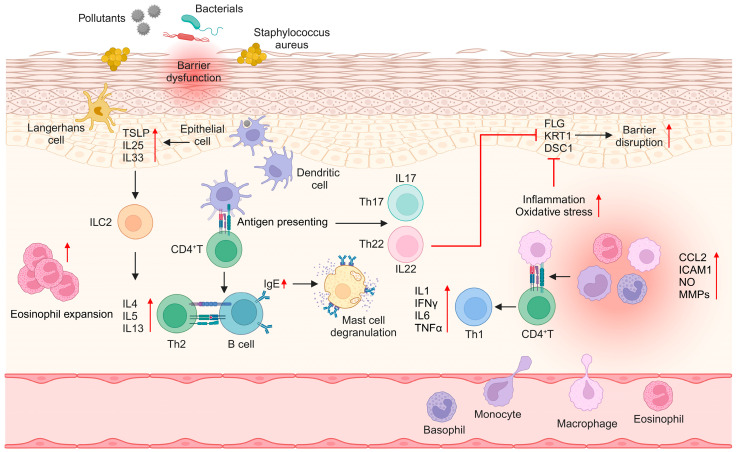
Pathogenesis of AD. AD is caused by genetic variations and repeated exposure to harmful substances including environmental pollutants, bacteria, and microbes. AD is developed by hypersensitivity or unbridled immune responses mediated by activated immune cells including eosinophils, mast cells, Langerhans cells, and T lymphocytes, and it becomes chronic and worsens due to skin barrier dysfunction and moisture loss. C-C motif chemokine ligand 2: CCL2; desmocollin 1: DSC1; filaggrin: FLG; intercellular adhesion molecule 1: ICAM1; interferon gamma: IFNγ; immunoglobulin E: IgE; interleukin: IL; type 2 innate lymphoid cell: ILC2; keratin 1: KRT1; matrix metalloproteinase: MMP; nitric oxide: NO; helper T: Th; tumor necrosis factor alpha: TNFα. Created with BioRender.com. Accessed on 5 November 2024.

AD is a prevalent, chronic inflammatory skin condition characterized by pruritus, erythema, and impaired skin barrier function, affecting up to 20% of children and 10% of adults globally [19,20]. The pathophysiology of AD is complex, multifactorial, and involves genetic predisposition, immune dysregulation, and environmental factors. Genetic mutations, such as those in the filaggrin gene, contribute to skin barrier dysfunction, whereas immune dysregulation is marked by an imbalance in Th2 and Th1 cytokines, promoting immunoglobulin (Ig) E-mediated hypersensitivity [20,21]. Environmental factors, including pollutants and allergens, further exacerbate this condition by triggering both innate and adaptive immune responses [21] (Figure 1). Traditional treatments for AD have included topical corticosteroids and emollients, which remain foundational but have limitations, particularly in moderate-to-severe cases that often require systemic therapy [22,23]. Recent advancements have introduced biological agents and small-molecule inhibitors targeting specific immunological pathways, such as monoclonal antibodies against IL4, IL13, and IL31 receptors, and Janus kinase (JAK) inhibitors, which offer more targeted mechanisms to modulate immune responses [22,23,24]. Biologics such as dupilumab, tralokinumab, and lebrikizumab have shown efficacy in reducing inflammation and achieving prolonged remission, suggesting their potential disease-modifying effects [25,26]. JAK inhibitors, such as baricitinib and upadacitinib can modify underlying disease mechanisms by simultaneously inhibiting multiple cytokine pathways [25,27]. Despite these advancements, there remains an unmet need for therapies that suppress symptoms and modify the underlying disease pathology, with ongoing research focusing on personalized medical approaches and the identification of biomarkers for tailored treatment strategies [22,28]. The evolving therapeutic landscape underscores the importance of continued research to optimize patient outcomes and address the significant burden that AD imposes on the quality of life and healthcare systems [20,23].

## 3. AhR in AD

**Figure 2:**
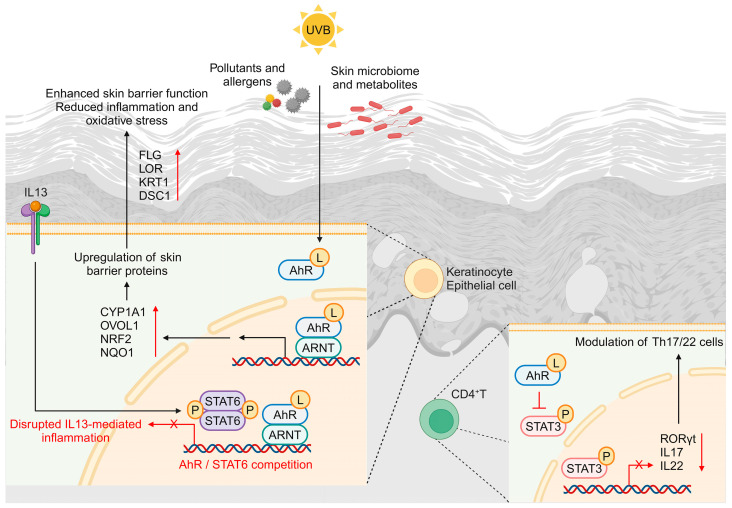
AhR signaling pathways as a therapeutic target for AD. AhR is activated by a ligand and is associated with various transcription factors or signaling pathways to regulate skin barrier function and suppress oxidative stress and inflammatory responses. Aryl hydrocarbon receptor: AhR; aryl hydrocarbon receptor nuclear translocator: ARNT; cytochrome P450 family 1 subfamily A member 1: CYP1A1; desmocollin 1: DSC1; filaggrin: FLG; intercellular adhesion molecule 1: ICAM1; interferon gamma: IFNγ; immunoglobulin E: IgE; interleukin: IL; type 2 innate lymphoid cell: ILC2; keratin 1: KRT1; ligand: L; loricrin: LOR; signal transducer and activator of transcription: STAT; matrix metalloproteinase: MMP; nitric oxide: NO; NAD(P)H quinone dehydrogenase 1: NQO1; nuclear factor erythroid 2-related factor 2: NRF2; ovo-like transcriptional repressor 1: OVOL1; retinoic acid-related orphan receptor gamma t: RORγt; helper T: Th; tumor necrosis factor alpha: TNFα; ultraviolet B: UVB. Created with BioRender.com. Accessed on 5 November 2024.

The AhR signaling pathway has emerged as a promising target for alleviating the symptoms of AD, a chronic inflammatory skin condition characterized by epidermal barrier dysfunction and immune dysregulation. AhR is a ligand-activated transcription factor that plays a crucial role in maintaining skin homeostasis and modulating immune responses. Studies have shown that AhR activation can reduce inflammation and improve skin barrier function, which is critical in managing AD symptoms [29,30] (Figure 2). Natural products, such as quercetin and ginsenosides, have shown the potential to exert anti-inflammatory effects by targeting various inflammatory cells and cytokines, further supporting the therapeutic potential of natural products in AD management [14]. Dysfunction of the AhR pathway in AD, characterized by increased AhR expression but reduced pathway activation, suggests a complex interplay between AhR and the inflammatory milieu in AD, necessitating further investigation into its therapeutic modulation [1]. Tapinarof, a topical AhR agonist, has shown promise in clinical trials, highlighting the potential of AhR-targeted therapies for AD [16,31]. Moreover, the crosstalk between AhR and IL13 signaling pathways in keratinocytes suggests that AhR activation can modulate IL13-driven responses, which are pivotal in AD pathogenesis [1,15]. Overall, the integration of natural products and AhR-targeted therapies offers a novel and promising approach to managing AD, and ongoing research is needed to fully elucidate the mechanisms and optimize therapeutic strategies [16,17].

## 4. Signaling Pathways Involved in AD Related to AhR

AhR is a crucial transcription factor that plays a significant role in skin physiology and immune regulation, particularly in AD. AhR is a ligand-activated receptor that modulates the expression of several genes that affect skin barrier integrity, immune responses, and inflammation. In AD, AhR signaling has emerged as a key pathway involved in disease progression, and its dysregulation is associated with impaired skin barrier function and heightened immune activity. Understanding the signaling mechanisms of AhR in AD offers insights into potential therapeutic strategies for this disease. A comprehensive summary of these concepts is provided in Table 1.

### 4.1. AhR-OVOL1 Signaling Pathway

AhR plays a significant role in the pathogenesis of AD by modulating the expression of genes involved in maintaining skin homeostasis and suppressing inflammation. In AD, the AhR pathway appears to be dysfunctional, as evidenced by increased AhR expression and decreased activation of its target genes, such as CYP1A1, in lesional skin compared to healthy skin [4]. OVO-like transcriptional repressor 1 (OVOL1), a direct transcriptional target of AhR, is essential for AhR barrier-promoting functions, and its deletion exacerbates AD-like inflammation [32]. The interaction between AhR and OVOL1 regulates the expression of skin barrier proteins such as filaggrin (FLG) and loricrin (LOR). This regulation is significant because mutations in FLG and dysregulation of OVOL1 are associated with AD, a condition characterized by skin barrier dysfunction and inflammation [33,34,35]. Activation of AhR by specific ligands, such as 6-formylindolo(3,2-*b*)carbazole (FICZ) and glyteer, has been shown to upregulate OVOL1 and subsequently increase FLG expression, which is often reduced in patients with AD [34]. Moreover, the AhR-OVOL1 axis is involved in modulating immune responses, as it can inhibit the expression of proinflammatory cytokines like IL33, which are elevated in AD [36]. The interplay between AhR and OVOL1 also extends to the regulation of other immune pathways such as IL13 signaling, which exacerbates AD symptoms by downregulating the OVOL1-FLG axis [37]. Furthermore, the AHR-OVOL1 pathway contributes to the maintenance of skin homeostasis by promoting the expression of genes involved in barrier function and immune regulation, thereby counteracting the typical inflammatory milieu typical of AD [32]. Overall, the AHR-OVOL1 signaling pathway represents a promising target for developing new treatments for AD, focusing on restoring skin barrier function and modulating immune responses.

### 4.2. AhR-Nuclear Factor Erythroid 2-Related Factor 2 (Nrf2) Pathway

The interaction between the AhR-Nrf2 pathway and signal transducer and activator of transcription (STAT) 3 in AD involves complex regulatory mechanisms that influence skin barrier function and immune responses. The AhR and Nrf2 pathways are crucial for maintaining skin homeostasis and mitigating oxidative stress, which are significant factors in the pathogenesis of AD. The strategy of AhR activation by ligands such as tapinarof has a potentiation in clinical trials for AD, as it enhances the expression of skin barrier proteins such as filaggrin, loricrin, and involucrin, thereby improving barrier function and reducing inflammation [26,27]. The AhR pathway is often dysregulated in AD, with increased AhR expression and reduced activation of its downstream targets, such as CYP1A1, suggests a dysfunctional pathway that fails to exert its full anti-inflammatory effects. The interplay between the Nrf2 and the AhR pathways is increasingly recognized as a significant factor in the pathogenesis and potential treatment of AD. Nrf2 and AhR are transcriptional regulators that modulate the expression of genes implicated in detoxification and antioxidative responses, which are essential for upholding dermal homeostasis and barrier integrity [38]. The restoration of AD-affected skin is partially facilitated through the antioxidative properties of Nrf2, which is activated downstream from AhR [39]. Furthermore, the AhR-Nrf2 axis is engaged in the modulation of oxidative stress, a prevalent characteristic in AD, by augmenting the synthesis of antioxidative enzymes [40]. The STAT3 pathway, activated by cytokines such as IL4 and IL13, contributes to barrier dysfunction by downregulating these genes, inducing oxidative stress and exacerbating inflammation [26,28]. There is a competitive interaction between the AhR-Nrf2 axis, which upregulates barrier function, and the IL13/IL4-JAK-STAT6/STAT3 axis, which downregulates it [28]. This interplay highlights the therapeutic potential of targeting the AhR-Nrf2 pathway to counteract the detrimental effects of the STAT3 pathway in AD and offers a novel approach for restoring skin barrier integrity and reducing inflammation.

### 4.3. Cytokines and Immune Response-Related Pathways

AhR plays a significant role in cytokine signaling pathways associated with AD, particularly through its interaction with various cytokines and immune responses. AhR influences the expression of several cytokines, including IL17, IL22, and IL33, which are crucial in the pathogenesis of AD. The IL33–IL37 axis, regulated by AhR, is particularly important, as IL33 promotes inflammation, whereas IL37 suppresses it, and their imbalance is implicated in AD pathogenesis [41]. AhR activation has been shown to modulate immune responses and improve skin barrier function, as demonstrated by the administration of Bojungikgi-tang, a traditional herbal formula widely used in northeast Asia, to AD mice, which reduced the levels of inflammatory markers and improved skin barrier integrity [29]. Furthermore, AhR signaling is involved in crosstalk with IL13, a cytokine that drives immune responses in AD, suggesting that AhR can attenuate IL13-induced effects in keratinocytes [1,15]. Despite its potential, AhR signaling is often dysregulated in AD, with increased expression and reduced pathway activation, as shown by the decreased expression of target genes such as CYP1A1 in lesional skin [4,8]. This dysregulation may contribute to the inflammatory milieu in AD because AhR is highly expressed in T cell subsets, including Th17 and Th22 cells, which are elevated in AD [42]. Overall, the AhR pathway is intricately linked to cytokine signaling in AD, influencing both immune responses and skin barrier functions, and represents a promising target for therapeutic interventions.

### 4.4. Microbiome and Its Metabolites Interaction Pathway

AhR plays a significant role in the pathogenesis of AD by interacting with the skin microbiome and immune pathways. The skin microbiome, particularly *Staphylococcus aureus*, can exacerbate AD by disrupting the skin barrier and promoting Th2 cytokine production, which can be modulated by AhR activation [43]. Therapeutic skin microbes, such as *Cutibacterium avidum*, can activate the AhR and Nrf2 pathways, restore skin barrier function, and reduce inflammation [43]. Additionally, tryptophan metabolites from skin microbiota, such as indole-3-aldehyde, can attenuate inflammation in AD through AhR activation, highlighting the role of the microbiome in modulating AhR pathways [44]. Similarly, Landemaine et al. showed that *Staphylococcus epidermidis*, recognized as an important member of the healthy skin microbiota, induced the AhR-OVOL1 axis and produced high quantities of indole-3-aldehyde and indole-3-lactic acid in healthy skin compared to AD-like skin [45]. Overall, the interaction of the AhR pathway with the microbiome and environmental factors underscores its potential as a therapeutic target in AD, with interventions such as AhR agonists showing promise in clinical trials.

### 4.5. Ultraviolet (UV) Light Pathway

AhR plays a significant role in the pathogenesis and treatment of AD, particularly upon exposure to ultraviolet (UV) light. UVB radiation, a component of sunlight, activates AhR, which then translocates to the nucleus to regulate gene expression, linking environmental stimuli to cellular responses [46,47]. This activation can modulate immune responses and enhance skin barrier integrity, potentially alleviating AD symptoms [33,48]. Phototherapy, which utilizes UV light, has been shown to exert therapeutic effects on AD by modulating AhR activity, highlighting its potential as a treatment strategy [30]. Furthermore, AhR activation by UVB can induce antioxidative pathways, such as AhR/Nrf2 signaling, which reduce oxidative stress and inflammation, further supporting its therapeutic potential in AD [16]. FICZ, a highly potent endogenous AhR ligand, has been shown to exert anti-inflammatory effects by downregulating FcεRI and upregulating indoleamine 2,3-dioxygenase in Langerhans cells, suggesting a feedback mechanism that could mitigate allergen-induced inflammation in AD [49]. Furthermore, FICZ restored the expression of FLG, a critical protein for skin barrier function, via the OVOL1 pathway, which is often compromised in AD due to genetic mutations. This restoration is crucial because FLG deficiency is a hallmark of AD, leading to an impaired skin barrier and increased susceptibility to allergens [34]. The formation of FICZ from tryptophan via light-independent pathways, including enzymatic processes involving Malassezia yeast, highlights its potential systemic role as an AhR agonist [50]. Collectively, phototherapy using UV light leads to the FICZ-mediated AhR activation that induces OVOL1-associated pathways, increases the production of tryptophan metabolite, and promotes the Nrf2-mediated antioxidative pathways, offering a multifaceted approach to modulate skin barrier function and immune responses.

### 4.6. Particulate Matter-Related Pathway

AhR plays a significant role in AD pathogenesis through its interaction with particulate matter (PM), which exacerbates skin inflammation and barrier dysfunction. PM, particularly PM2.5, induces skin barrier dysfunction and inflammation by upregulating Th17 cell-related genes and AhR-regulated genes, such as IL6 and IL36G, in human skin tissue [39]. AhR, a transcription factor expressed in all skin cells, is activated by pollutants such as polycyclic aromatic hydrocarbons, leading to deleterious effects on keratinocytes and fibroblasts, which are central to the skin response to pollutants [40]. In mouse models, PM exposure has been linked to increased skin severity scores and epidermal thickness, highlighting the role of AhR in mediating these effects through the differential expression of genes related to skin barrier integrity and the immune response [41]. Furthermore, AhR activation by air pollutants induces the expression of neurotrophic factors such as artemin, contributing to epidermal hyperinnervation and hypersensitivity to pruritus, which are characteristics of AD [42]. The AhR pathway also modulates the balance between regulatory and effector T cells, thereby influencing the immune responses in AD [43]. Overall, AhR serves as a critical mediator linking air pollution to the exacerbation of AD, highlighting its potential as a therapeutic target for managing this condition.

## 5. Natural Products as Potential AhR Modulators

**Figure 3:**
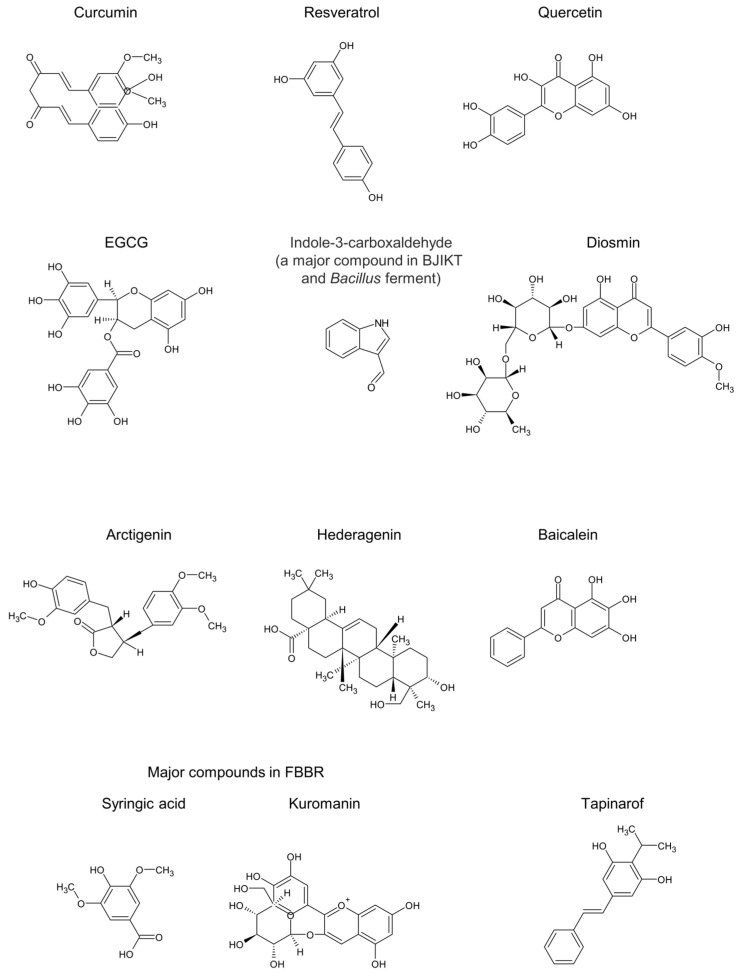
Chemical structures of potential AhR-modulating natural products. Indole-3-carboxaldehyde is a major compound in both Bojungikgi-tang (BJIKT) and *Bacillus* ferment. Syringic acid and kuromanin are major compounds in fermented blueberry and black rice (FBBR). EGCG, Epigallocatechin gallate. The chemical structures were generated using ChemDraw (ver. 23.1.1).

Natural products have emerged as promising AhR modulators for treating AD, a chronic inflammatory skin condition. Several studies have identified various natural compounds that can modulate AhR activity, which is crucial for managing skin inflammation and maintaining skin barrier function. These findings underscore the therapeutic potential of natural AhR modulators in managing AD, offering a promising alternative to conventional treatments with fewer side effects and improved patient compliance [51]. Overall, the exploration of natural products as AhR modulators provides a valuable avenue for the development of effective and safe treatments for AD. A comprehensive summary of these findings is provided in Table 2. In addition, the chemical structures of these compounds are shown in Figure 3.

### 5.1. Curcumin

Curcumin, a bioactive compound derived from turmeric, has shown the potential to alleviate AD through various mechanisms, including the modulation of AhR. The anti-inflammatory properties of curcumin have been demonstrated in several studies, wherein it effectively reduced the levels of inflammatory cytokines and chemokines in AD models. For instance, bisdemethoxycurcumin, a curcumin derivative, was found to significantly improve symptoms in 2,4-dinitrochlorobenzene (DNCB)-induced AD in mice by inhibiting inflammatory pathways such as mitogen-activated protein kinase (MAPK) and nuclear factor kappa B (NFκB), which are crucial in the pathogenesis of AD [52]. Additionally, curcumin has been shown to ameliorate ovalbumin-induced AD in mice by suppressing Th2 cytokines and restoring the redox balance, further supporting its role in modulating immune responses in AD. Curcumin may contribute to the therapeutic effects in AD through skin homeostasis and immune modulation by activating AhR. The interaction of curcumin with AhR has been explored in the context of its antiaging effects, demonstrating both AhR-dependent and -independent pathways, suggesting a complex mechanism of action that could be beneficial for AD treatment [53]. Furthermore, the efficacy of curcumin in reducing IL13 levels and promoting re-epithelialization in rats with acetone-induced dermatitis highlights its potential as a therapeutic agent for AD [54]. Collectively, these findings suggested that curcumin, through its interaction with AhR and other pathways, is a promising natural therapeutic option for the management of AD.

### 5.2. Resveratrol

Resveratrol, a naturally occurring polyphenol, has shown promise in alleviating AD through its interaction with AhR. The therapeutic potential of resveratrol in AD is attributed to its anti-inflammatory properties and its ability to modulate key pathways involved in the pathogenesis of AD. Studies have demonstrated that resveratrol can attenuate mast cell activation, reduce perivascular cell infiltration, and decrease the production of inflammatory chemokines, which are critical in the early phases of AD [55]. Additionally, resveratrol has been shown to ameliorate histological changes in AD-like lesions by reducing epithelial thickness and expression of proinflammatory cytokines such as IL25, IL33, and thymic stromal lymphopoietin (TSLP), while also decreasing epithelial apoptosis. The modulation of AhR by resveratrol is particularly significant because AhR plays a crucial role in epidermal differentiation and skin barrier function. Antioxidative phytochemicals, such as resveratrol, can activate the AhR-OVOL1 pathway, promoting the expression of differentiation molecules, such as FLG, which are essential for repairing skin barrier disruption in AD [33]. Furthermore, the ability of resveratrol to downregulate proinflammatory cytokines and upregulate proteins such as FLG and transglutaminase suggests its efficacy in improving skin barrier integrity and reducing inflammation [56]. The potential of resveratrol as an AhR ligand is supported by quantitative structure–activity relationship studies, which highlight its structural properties that favor AhR binding, thereby enhancing its therapeutic effects [57]. Overall, the multifaceted action of resveratrol on AhR signaling and its anti-inflammatory effects make it a promising candidate for the treatment of AD, offering a natural alternative to conventional therapies with fewer adverse effects.

### 5.3. Quercetin

Quercetin, a naturally occurring flavonoid, has shown the potential to alleviate AD through the modulation of AhR. Quercetin exhibits strong antioxidant and anti-inflammatory properties, which are beneficial for treating skin disorders such as AD [58,59]. In a study involving an AD mouse model, quercetin treatment significantly reduced skin lesions and inflammation by modulating the high mobility group box 1/receptor advanced glycation end products/NFκB signaling pathway and enhancing the expression of the Nrf2 protein [58]. This suggests that quercetin can mitigate the inflammatory responses, which are key aspects of AD pathogenesis. Furthermore, quercetin, along with other polyphenols, such as resveratrol and curcumin, has been identified as an indirect activator of AhR. These compounds inhibit the metabolic degradation of the endogenous AhR ligand FICZ, thereby prolonging AhR activation [60]. Thus, the ability of quercetin to modulate AhR activity, combined with its anti-inflammatory effects, makes it a promising natural agent for the treatment of AD.

### 5.4. Green Tea Extract and Epigallocatechin-3-Gallate (EGCG)

Green tea extract and its active component, epigallocatechin-3-gallate (EGCG), have shown potential in alleviating AD through the modulation of the AhR pathway. EGCG, a major catechin found in green tea, has demonstrated significant anti-inflammatory effects in AD models. For instance, EGCG nanoparticles have been shown to ameliorate AD symptoms in mice by reducing skin thickness, dermatitis scores, and inflammatory cytokine levels, while inhibiting necroptosis, a form of programmed cell death associated with inflammation [61]. The mechanism by which green tea extract and EGCG exert their effects may involve modulation of the AhR pathway, which has been implicated in the regulation of immune responses and skin barrier integrity in AD [62]. Overall, green tea extract and its major compound EGCG hold promise in AD treatment regimens, particularly through their interaction with the AhR pathway, offering a natural and effective approach to managing this chronic inflammatory skin condition.

### 5.5. Bojungikgi-Tang (BJIKT)

Bojungikgi-tang (BJIKT), a traditional herbal formula, has been extensively studied for its therapeutic potential against various medical conditions, highlighting its role as a natural product with diverse applications. BJIKT has shown promise as a natural product for alleviating AD, primarily through its effects on the skin barrier function and immune response. Studies have demonstrated that BJIKT effectively improves AD symptoms in mouse models by modulating the AhR pathway, which is crucial for maintaining skin barrier integrity and regulating immune responses. In particular, BJIKT administration led to a reduction in neutrophil and eosinophil counts, decreased serum IgE levels, and lowered IL4 and IFNγ levels in splenocytes, which are indicative of a reduced inflammatory response [29]. Additionally, BJIKT decreased epithelial skin thickness and transepidermal water loss (TEWL) while reversing the downregulation of skin barrier genes, thereby enhancing the protective function of the skin. The major compound in BJIKT identified as influencing AD is indole-3-carboxaldehyde, an AhR ligand [29]. The herbal formulation also significantly altered the expression of AhR target genes such as AhR, AhR repressor, CYP1A1, and CYP1B1, which were negatively correlated with immune cell subtypes, including CD4^+^ and CD8^+^ T cells and macrophages, suggesting systemic modulation of immune activity [29]. The potential of natural products such as BJIKT in treating AD is further supported by broader research on natural extracts, which highlights their ability to mitigate inflammation, improve skin barrier function, and reduce the adverse effects associated with conventional treatments such as corticosteroids [17,63,64]. Furthermore, other herbal formulations, such as Chijabyukpi-tang and Gagambojungikgi-tang, have also demonstrated anti-inflammatory effects in AD models by modulating cytokine production and promoting wound healing [65,66]. Overall, BJIKT and similar natural products offer promising avenues for AD management, emphasizing the need for further research to fully elucidate their mechanisms and optimize their therapeutic potential.

### 5.6. Diosmin

Diosmin, a flavonoid with notable anti-inflammatory and antioxidant properties, has shown the potential to alleviate AD through its interaction with AhR. Diosmin and its aglycone form, diosmetin, are natural dietary AhR agonists [67]. Activation of AhR by diosmin can lead to increased transcription of CYP1A1, a gene involved in detoxification processes, suggesting a role in maintaining skin homeostasis and reducing oxidative stress [67]. Diosmin, an AhR agonist, increased and restored the expression of skin barrier proteins (FLG and LOR), which were suppressed by Th2 cytokines. Additionally, diosmin directly binds to AhR, promotes its nuclear translocation, inhibits STAT3 phosphorylation, and increases epidermal thickness [68]. Furthermore, diosmetin, a glycosidic form of diosmin derived from *Lobelia chinensis*, has been shown to improve skin barrier function by upregulating the serine peptidase inhibitor Kazal type 5/lympho-epithelial Kazal-type-related inhibitor, which inhibits serine protease activity, thereby preventing skin barrier damage and normalizing immune responses in AD models [69]. These findings collectively suggest that through its action on AhR, diosmin holds promise as a natural therapeutic agent for managing AD by improving skin barrier function and modulating immune responses.

### 5.7. Coal Tar and Glyteer

Coal tar and glyteer, both natural products, have historically been used to alleviate AD by modulating the AhR, a key player in skin barrier function and immune response regulation. Coal tar, a complex mixture of hydrocarbons, has been shown to activate AhR, leading to the induction of epidermal differentiation and restoration of skin barrier proteins such as filaggrin, which are often downregulated in AD owing to Th2 cytokine activity [70]. This activation also involves the Nrf2 pathway, which helps mitigate oxidative stress, a contributor to the inflammatory milieu in AD [39]. Glyteer, derived from soybean tar, similarly activates AhR and has been demonstrated to impair IL4/STAT6 signaling in dendritic cells, reducing the production of Th2-attracting chemokines CCL17 and CCL22, thereby potentially alleviating AD symptoms [71,72]. Despite their efficacy, the use of coal tar and glyteer is limited by their unpleasant odor and appearance, prompting the development of alternatives such as tapinarof, a synthetic AhR agonist that offers similar therapeutic benefits without these drawbacks [70,73]. The therapeutic effects of coal tar and glyteer underscore the importance of AhR as a pharmacological target and offer insights into the development of new treatments for AD that harness the role of AhR in skin homeostasis and inflammation control [48]. Although the carcinogenic potential of coal tar is a concern, epidemiological studies suggest that its topical use does not pose significant risks, making it a viable option for treating chronic dermatological conditions [74]. Overall, the modulation of AhR by coal tar and glyteer represents a promising strategy for AD treatment, with ongoing research aimed at optimizing these therapies for better patient outcomes [74,75]. Furthermore, integration of these compounds into combination therapies may enhance their efficacy, potentially leading to more comprehensive management strategies for patients with AD.

### 5.8. A Mixture of Arctigenin, Hederagenin, and Baicalein

A mixture containing arctigenin, hederagenin, and baicalein shows promise in alleviating AD by modulating AhR. This combination, derived from Forsythiae Fructus, Lonicerae Flos, and Scutellariae Radix, has demonstrated significant anti-inflammatory and antiatopic effects in experimental models. In a study involving DNCB-induced mice, topical application of this mixture reduced symptoms of AD, such as epidermal thickness and mast cell infiltration, and decreased the levels of proinflammatory cytokines like TNFα, IFNγ, and IL4, as well as serum IgE levels [76]. Natural AhR ligands, such as those found in the mixture, can regulate immune responses by promoting the differentiation of regulatory T cells and reducing proinflammatory Th17 cells, thus offering a therapeutic avenue for inflammatory diseases such as AD [77]. Furthermore, AhR activation by antioxidant phytochemicals can enhance epidermal differentiation and repair skin barrier disruptions, which is crucial for the management of AD [33]. Overall, the integration of arctigenin, hederagenin, and baicalein into therapeutic strategies for AD could leverage the regulatory capabilities of AhR to provide effective and safer treatment options.

### 5.9. Microbial Metabolites and Bacillus Ferment

Microbial metabolites, particularly those derived from tryptophan metabolism, are promising natural products for alleviating AD by modulating AhR. Studies have demonstrated that certain skin and gut microbiota can metabolize tryptophan to indole derivatives, which are potent agonists for AhR. For instance, skin microbiota-derived indole-3-aldehyde has been shown to significantly reduce skin inflammation in AD by activating AhR, which in turn inhibits the expression of proinflammatory cytokines such as TSLP [44]. Similarly, *Bifidobacterium longum* and *Limosilactobacillus reuteri*, gut bacteria known for their ability to produce indole derivatives, such as indole-3-carbaldehyde and indole lactic acid, have been reported to alleviate AD symptoms through the gut–skin axis by modulating immune responses via AhR activation. These metabolites suppress aberrant Th2 immune responses and reshape the gut microbiota composition, further enhancing their therapeutic potential [78]. The role of the AhR in mediating these effects is underscored by studies showing that the benefits of these microbial metabolites are negated by AhR antagonists, highlighting the central role of the receptor in therapeutic mechanisms [44,78]. Additionally, modulation of AhR by other microbial metabolites, such as quinolinic acid, has been shown to regulate inflammatory pathways such as the NLR family pyrin domain-containing 3 inflammasome, further supporting the potential of targeting AhR in AD treatment [79]. *Bacillus* fermentation, particularly when involving strains such as *Bacillus amyloliquefaciens*, has shown potential for alleviating AD by modulating immune responses and possibly interacting with the AhR pathway. In a study using a soybean product fermented with *Bacillus amyloliquefaciens*, significant improvement in AD symptoms was observed in a mouse model. This was attributed to the suppression of mast cell infiltration, reduction in IgE expression, and decreased production of cytokines such as IL4 and IL31, which are involved in the inflammatory response associated with AD [80]. Collectively, these findings suggest that microbial metabolites and *Bacillus* fermentation, through their interactions with AhR, offer a promising avenue for the development of novel therapeutic strategies for managing AD.

### 5.10. Fermented Blueberry and Black Rice (FBBR)

Fermented blueberry and black rice (FBBR) have emerged as promising modulators of the AhR pathway, which has been implicated in the pathogenesis of AD and its exacerbation by PM exposure. AhR is a transcription factor that mediates various biological responses, including detoxification and immune regulation. It is activated by environmental pollutants such as PM2.5, which can worsen AD by promoting inflammation and oxidative stress [81,82]. Studies have shown that syringic acid and kuromanin are the primary compounds found in FBBR, containing *Lactobacillus plantarum* MG4221, can mitigate PM2.5-induced skin inflammation by modulating the MAPK/NFκB pathways, reducing proinflammatory cytokines such as IL1β, IL6, and IL8, and enhancing skin barrier proteins such as FLG and involucrin [83]. This suggests that FBBR may counteract the deleterious effects of PM on the skin by influencing AhR pathways, which are crucial for maintaining skin homeostasis and immune balance [30,82]. Furthermore, FBBR demonstrated neuroprotective effects against PM2.5-induced inflammation, highlighting its potential as a nutraceutical agent with broad health benefits beyond skin conditions [84]. Overall, integration of FBBR into dietary or topical applications could provide a novel approach for managing AD, particularly in environments with high PM exposure, by leveraging its ability to modulate key inflammatory and barrier-related pathways via AhR activation.

### 5.11. Tapinarof

Tapinarof, a first-in-class, nonsteroidal topical AhR agonist, has emerged as a promising therapeutic agent for AD owing to its ability to modulate immune responses and improve skin barrier function. Tapinarof has demonstrated significant efficacy in clinical trials, improving skin barrier function by increasing stratum corneum hydration and reducing TEWR, which are critical for managing AD [85]. The mechanism of action of tapinarof involves the downregulation of proinflammatory Th2 cytokines and upregulation of skin barrier components, which collectively contribute to its therapeutic effects [86]. Additionally, tapinarof has been shown to reduce oxidative stress and modulate T cell function, specifically by decreasing IL13 and IL17a levels in tissue-resident T cells, which have been implicated in the pathogenesis of AD and psoriasis. This modulation of cytokine production and T cell metabolism suggests a novel mechanism by which tapinarof exerts its anti-inflammatory effects [87]. The therapeutic potential of tapinarof is further supported by its favorable safety profile with minimal systemic exposure and adverse effects, making it suitable for long-term use in both adults and children [88]. Overall, tapinarof represents a significant advancement in the treatment of AD, offering a novel approach that leverages modulation of the AhR pathway to achieve clinical efficacy and improve patient outcomes.

### 5.12. Systematic Analysis of Natural Products as AhR Modulators in AD

These natural products demonstrate varied mechanisms of action, encompassing the attenuation of Th2 cytokines, diminution of inflammatory mediators, enhancement of skin barrier proteins, and modulation of oxidative stress. Direct AhR activators are agents that directly associate with and stimulate the AhR, resulting in numerous advantageous effects in AD treatment [89]. Illustrations include curcumin, resveratrol, diosmin, coal tar, and glyteer. These modulators exhibit extensive advantages across all classifications, including Th2 attenuation, inflammatory cytokine diminution, enhancement of skin barrier functionality, and reduction in oxidative stress. Indirect AhR modulators affect AhR activity through indirect mechanisms, such as impeding the degradation of endogenous AhR ligands [90]. Quercetin and epigallocatechin gallate are representative examples of this category. While efficacious in Th2 attenuation, inflammatory cytokine diminution, and oxidative stress reduction, they may not directly enhance skin barrier functionality. Complex action modulators are herbal formulations or amalgamations of compounds that operate via multiple pathways, including AhR modulation. BJIKT and combinations of arctigenin, hederagenin, and baicalein serve as examples. These products manifest effects on Th2 attenuation, inflammatory cytokine diminution, and enhancement of skin barrier functionality. Microbial-derived AhR modulators comprise metabolites from cutaneous and gastrointestinal microbiota that activate AhR and influence the gut–skin axis [91]. Instances include Bacillus ferment, tryptophan metabolites, and FBBR. These compounds primarily concentrate on Th2 attenuation and inflammatory cytokine diminution, modulating the gut–skin axis and reshaping gut microbiota composition. Synthetic AhR agonists, such as tapinarof, are compounds formulated based on natural AhR modulators. They demonstrate comprehensive benefits akin to direct activators, providing targeted AhR stimulation with optimized efficacy and safety profiles. These natural substances demonstrate varied mechanisms of action, encompassing the attenuation of Th2 cytokines, diminution of inflammatory mediators, enhancement of skin barrier proteins, and modulation of oxidative stress. The multifaceted actions of these compounds on AhR signaling and their anti-inflammatory effects render them promising candidates for AD treatment, potentially offering more comprehensive management strategies for this chronic inflammatory dermatosis. A thorough summary of these findings is provided in Table 3.

## 6. Conclusions

AD is a complex disease requiring a multifaceted treatment approach. AhR plays a critical role in regulating immune responses and maintaining the skin barrier function, making it a promising target for therapeutic intervention. Natural products such as BJIKT and indole-3-lactate have potential as AhR modulators that may alleviate AD symptoms and improve the quality of life of patients. AhR is a pivotal transcription factor that influences both immune response and skin barrier integrity. This review highlights the significant potential of natural products as modulators of AhR activity, suggesting that they may not only alleviate symptoms but also promote a more sustainable approach to AD management. The interplay among AhR signaling, environmental factors, and skin microbiome underscores the complexity of AD pathogenesis and treatment. Integrating natural therapies with conventional treatments is a promising approach to enhance patient outcomes and overall skin health. Further research should focus on elucidating the mechanisms of action of these natural products, exploring their synergistic effects on microbiome interactions, and considering the genetic predispositions to develop personalized medicinal approaches for AD management.

## Figures and Tables

**Table 1:** Key signaling pathways associated with AhR in AD.

Signaling Pathway	Key Features	Impact on AD Pathogenesis	Therapeutic Potential	References
AhR-OVOL1 Pathway	Regulates skin barrier proteins (FLG, LOR); modulates inflammation; OVOL1 essential for barrier integrity	Promotes skin barrier maintenance; dysfunction linked to AD inflammation and reduced target gene activation	Target for therapies enhancing OVOL1 and FLG expression; reduces AD symptoms	[32,33,34,35,36,37]
AhR-Nrf2 Pathway	Enhances skin barrier proteins; mitigates oxidative stress; interacts with STAT3/IL13 pathways	Counteracts STAT3-induced barrier dysfunction; enhances antioxidative responses and reduces inflammation	Potential for therapies like tapinarof to activate AhR and Nrf2; improves barrier function	[26,27,28,38,39,40]
Cytokines and Immune Responses	Modulates IL17, IL22, IL33; balances inflammatory and anti-inflammatory cytokine responses	Involved in immune regulation and inflammatory response; dysregulation linked to elevated AD cytokine levels	Therapeutic focus on cytokine modulation; attenuates inflammatory pathways in AD	[1,4,8,15,29,41,42]
Microbiome Interaction Pathway	Influences skin microbiome; tryptophan metabolites activate AhR for barrier and immune modulation	Modulates immune response via microbiome metabolites; reduces inflammation and enhances barrier function	Interventions targeting AhR-activating microbiome components could benefit AD treatment	[43,44,45]
UV Light Pathway	Activated by UVB; enhances skin barrier; triggers antioxidative pathways; links to FICZ as a natural AhR ligand	Supports phototherapy for AD; modulates gene expression for skin integrity and inflammation control	Supports the use of phototherapy and AhR-targeting compounds such as FICZ	[16,30,33,34,46,47,48,49,50]
Particulate Matter Pathway	Activated by PM2.5/PAHs; induces Th17-related inflammation; impacts keratinocytes and fibroblasts	Links pollution to increased AD symptoms; exacerbates barrier dysfunction and immune activation	AhR antagonists or protectants could reduce pollution-induced AD exacerbation	[38,39,40,41,42]

Atopic dermatitis: AD; aryl hydrocarbon receptor: AhR; 6-formylindolo(3,2-*b*)carbazole: FICZ; filaggrin: FLG interferon gamma: IFNγ; interleukin: IL; ligand: L; loricrin: LOR; signal transducer and activator of transcription: STAT; nuclear factor erythroid 2-related factor 2: Nrf2; ovo-like transcriptional repressor 1: OVOL1; polycyclic aromatic hydrocarbon: PAH; particulate matter: PM; helper T: Th; ultraviolet: UV.

**Table 2:** Natural products as AhR modulators in AD treatment.

Natural Product	Mechanism of Action	Effects on AD	References
Curcumin	Modulates AhR; anti-inflammatory; suppresses Th2 cytokines; restores redox balance	Improves symptoms in AD models; reduces IL13 levels; promotes re-epithelialization	[52,53,54]
Resveratrol	Activates AhR; anti-inflammatory; reduces mast cell activation and perivascular cell infiltration	Improves skin barrier integrity; reduces inflammation and histological changes in AD lesions	[33,55,56,57]
Quercetin	Activates AhR; anti-inflammatory and antioxidant properties; prolongs AhR activation by inhibiting degradation of FICZ	Reduces skin lesions and inflammation by modulating NFκB and Nrf2 signaling pathways	[58,59,60]
Green Tea Extract (EGCG)	Modulates AhR; anti-inflammatory; inhibits necroptosis	Reduces dermatitis scores, skin thickness, and cytokine levels in AD models	[61,62]
Bojungikgi-tang	Activates AhR; reduces immune cell counts; decreases IL4, IFNγ, and serum IgE	Improves skin barrier function; reduces transepidermal water loss	[29,63,64,65,66]
Diosmin	Activates AhR; increases transcription of detoxification gene CYP1A1; inhibits STAT3 phosphorylation	Improves skin barrier proteins (FLG, LOR); enhances skin homeostasis	[67,68,69]
Coal Tar and Glyteer	Activates AhR; induces epidermal differentiation; modulates Nrf2	Restores skin barrier proteins; reduces Th2 cytokine activity; alleviates AD symptoms	[39,70,71,72,73,74,75]
Arctigenin, Hederagenin, Baicalein	Activates AhR; anti-inflammatory; derived from multiple herbal sources	Reduces epidermal thickness, mast cell infiltration, and cytokine levels in AD models	[33,76,77]
Microbial Metabolites and Bacillus Ferment	Metabolites activate AhR; modulate gut–skin axis; reduce Th2 immune responses	Decrease skin inflammation and cytokine levels; improve gut microbiota composition	[44,78,79,80]
Fermented Blueberry and Black Rice	Modulates AhR; reduces PM2.5-induced inflammation; improves skin barrier proteins	Mitigates skin inflammation; reduces proinflammatory cytokines	[30,81,82,83,84]
Tapinarof	Nonsteroidal AhR agonist; improves skin barrier function; reduces Th2 cytokines	Increases stratum corneum hydration; reduces transepidermal water loss	[85,86,87,88]

Atopic dermatitis: AD; aryl hydrocarbon receptor: AhR; cytochrome P450 family 1 subfamily A member 1: CYP1A1; 6-formylindolo(3,2-*b*)carbazole: FICZ; filaggrin: FLG interferon gamma: IFNγ; immunoglobulin E: IgE; interleukin: IL; ligand: L; loricrin: LOR; signal transducer and activator of transcription: STAT; nuclear factor erythroid 2-related factor 2: Nrf2; particulate matter: PM; helper T: Th.

**Table 3:** Classification of AhR modulators in AD treatment: mechanisms and effects.

Group	Th2 Suppression	Inflammatory Cytokine Reduction	Skin Barrier Function Improvement	Oxidative Stress Reduction	Compounds
Direct AhR Activators	✓	✓	✓	✓	Curcumin, Resveratrol, Quercetin, Diosmin, Coal tar and Glyteer
Indirect AhR Activators	✓	✓	-	✓	Green tea extract (EGCG)
Complex Action Products	✓	✓	✓	-	Bojungikgi-tang (BJIKT), Arctigenin/Hederagenin/Baicalein mixture
Microbial Metabolites	✓	✓	-	-	Bacillus ferment, Tryptophan metabolites, Fermented blueberry and black rice (FBBR)
Synthetic AhR Agonists	✓	✓	✓	✓	Tapinarof

## Data Availability

Not applicable.
